# Development and testing of two tools to assess point-of-sale food and beverage marketing to children in restaurants

**DOI:** 10.1017/S1368980024000983

**Published:** 2024-05-06

**Authors:** Leia M Minaker, Patrycia Menko, David Olona

**Affiliations:** School of Planning, Faculty of Environment, University of Waterloo, Waterloo, ON N2L 3G1, Canada

**Keywords:** Marketing to children, Restaurants, Tool development and testing, Canada

## Abstract

**Objective:**

To describe the development and testing of two assessment tools designed to assess exterior (including drive-thru) and interior food and beverage marketing in restaurants with a focus on marketing to children and teens.

**Design:**

A scoping review on restaurant marketing to children was undertaken, followed by expert and government consultations to produce a draft assessment tool. The draft tool was mounted online and further refined into two separate tools: the Canadian Marketing Assessment Tool for Restaurants (CMAT-R) and the CMAT-Photo Coding Tool (CMAT-PCT). The tools were tested to assess inter-rater reliability using Cohen’s Kappa and per cent agreement for dichotomous variables, and intra-class correlation coefficients (ICCs) for continuous or rank-order variables.

**Setting:**

Waterloo, Ontario, Canada.

**Participants:**

Restaurants of all types were assessed using the CMAT-R (*n* 57), and thirty randomly selected photos were coded using the CMAT-PCT.

**Results:**

The CMAT-R collected data on general promotions and restaurant features, drive-thru features, the children’s menu and the dollar/value menu. The CMAT-PCT collected data on advertisement features, features considered appealing to children and teens, and characters. The inter-rater reliability of the CMAT-R tool was strong (mean per cent agreement was 92·4 %, mean Cohen’s κ = 0·82 for all dichotomous variables and mean ICC = 0·961 for continuous/count variables). The mean per cent agreement for the CMAT-PCT across items was 97·3 %, and mean Cohen’s κ across items was 0·91, indicating very strong inter-rater reliability.

**Conclusions:**

The tools assess restaurant food and beverage marketing. Both showed high inter-rater reliability and can be adapted to better suit other contexts.

Globally^(1,2)^, and in Canada^(3,4)^, children are exposed to a high level of unhealthy food and beverage marketing via multiple media channels. Food and beverage marketing to children (M2K) has negative impacts on their health and nutrition^(1,2,5,6)^, which is why over a dozen countries have implemented M2K restrictions^(7)^. Of the statutory regulations that exist, television M2K is the most frequently restricted medium, followed by M2K in schools^(7)^. In 2016, Chile implemented arguably the most comprehensive regulations restricting M2K. Chile’s Law of Nutrition Composition of Food and Advertising prohibits advertising (including point-of-sale advertising) of foods and beverages high in calories, saturated fat, sugar and sodium to children under the age of 14 years^(8)^. Other countries, including Canada, are currently in the process of establishing M2K regulations. Despite the growing number of regulations, limited resources and methodological challenges are important barriers to monitoring compliance^(9)^. Ongoing M2K monitoring is especially critical during policy implementation to ensure compliance and policy enforcement^(10)^.

Food industry responses to existing and forthcoming M2K regulations are still not fully understood, although awareness of the huge impact of commercial determinants of health and the food industry’s role in determining health is growing^(11)^. A recent systematic review found that despite a low certainty of evidence in the research to date, policies (especially mandatory policies) can effectively limit food marketing to children^(5)^. However, food industry responses to limitations in broadcast media and educational marketing channels are yet to be fully determined^(7)^. It seems reasonable to expect that if point-of-sale marketing is exempt from national policies to restrict M2K, food and beverage companies would turn their attention to remaining marketing avenues and invest more heavily in this type of marketing. This has certainly been the case with tobacco advertising: as each form of promotion has been banned, tobacco firms use other marketing strategies to continue communicating brand imagery^(12–14)^. For example, when tobacco product advertising was banned, sponsorship expenditures increased considerably, and when tobacco sponsorship was prohibited, the tobacco industry invested more heavily in point-of-sale strategies^(12,14)^. Several studies have documented food industry marketing practice in response to regulation (e.g. how commercial breastmilk substitutes evolved in response to civic action and policy implementation^(15)^, or the lack of transparency in food industry policies related to marketing to children^(16)^). However, given that current policy focuses on M2K in television, print and other media, it is important to monitor how point-of-sale marketing will evolve over time in response to regulations.

While M2K literature has expanded rapidly over the past decade, most M2K research to date has focused on digital, radio and television media, as well as M2K in specific settings (e.g. schools and recreation facilities). One of the current gaps in M2K research is the extent to which children are exposed to food marketing within restaurants^(3,4)^.

Restaurants are particularly important settings in which point-of-sale M2K can be monitored. Globally, a high proportion of children and youth frequently consume restaurant foods. Of thirty-two countries and over 105 000 participants aged 12–15 years in the Global School-based Student Health Survey, 53·5 % consumed fast food at least once per week, ranging from a low of 27 % in Cambodia to 80 % in Thailand^(17)^. A more recent study using data from fifty-four countries participating in the same survey found that 55 % of adolescents consumed fast food at least once per week, and 10 % did so between 4 and 7 d per week^(18)^. In the USA, restaurant spending has accounted for over half of total food expenditures since 2008^(19)^, with the exception of food-away-from-home spending decreasing between 2019 and 2020^(18)^ to just under half of expenditures (largely due to coronavirus disease 2019 pandemic-related restrictions in restaurants). In Canada in 2019, about a quarter of food expenditures (27 %) were due to restaurant spending^(20)^, and the fast food restaurant industry grew an average of 2·5 % between 2015 and 2020^(21)^, experiencing just over an 8 % market growth in 2022^(22)^. In 2019, 65 % of children 1–5 years old, 71 % of children 6–12 years old and 75 % of adolescents ate restaurant food at least once in the previous week^(23)^. Children’s meals in Canada^(24)^ tend to be of poor nutritional quality, particularly high in sodium and calories. It is unsurprising that fast food intake is associated with excess weight gain^(25)^ and poorer diet quality^(26)^ among children. Despite this, only two Canadian studies to date have assessed food marketing in restaurants^(27)^.

Many food environment assessment tools have been developed and tested for reliability and validity^(28)^, but few have focused on M2K in restaurants. In 2021, Cohen and colleagues^(29)^ published the Environmental Assessment Tool (EAT), adapted from the Nutrition Environment Measures Survey – Restaurants (NEMS-R)^(30)^. The EAT emphasises M2K strategies (e.g. posters, table tents and verbal prompts) in quick-service restaurants. The EAT was developed exclusively for quick-service restaurants and excluded data from drive-thrus and exterior advertisements. The Bridging the Gap Fast Food Observation Form^(31,32)^ was also designed to explicitly assess quick-service restaurants. Existing tools primarily focus on the nutritional quality of children’s menus, the price of healthier options relative to less healthy options and promotions (e.g. posters and toys with meal purchase). To date, no assessment tool has been developed to assess both interior and exterior (including drive-thrus) M2K designed for a variety of restaurant types. Given that drive-thrus may account for up to two-thirds of fast food purchases^(33)^ and that pandemic-related increases in drive-thru use by customers are anticipated to be maintained in the future above pre-pandemic levels^(34)^, a comprehensive tool capturing interior and exterior policy-relevant M2K across restaurant types is needed to support M2K monitoring.

The current study describes the development and testing of an assessment tool (the Canadian Marketing Assessment Tool for Restaurants: CMAT-R) and an associated Photo Coding Tool (the CMAT-PCT) specifically designed to assess exterior (including drive-thru) and interior marketing in restaurants in Canada, with a focus on marketing to children. Of note, the impact of M2K is a function of both exposure (e.g. reach, frequency and impact of the message) and power (content, design and execution)^(35)^. The CMAT-R and CMAT-PCT are designed to address both potential exposure (e.g. frequency of different types of advertising strategies) and power (content and design of interior and exterior advertisements). The CMAT-R was designed in consultation with Health Canada and marketing experts to be relevant to the Canadian policy context, with the ultimate goal of supporting policy monitoring and implementation in these settings. Although the tool was designed for the Canadian context, it may be also relevant for other jurisdictions considering point-of-sale M2K restrictions. The tool also aligns with the World Health Organization’s framework for implementing recommendations on M2K, in particular that food and beverage marketing policy frameworks ‘should include a monitoring system to ensure compliance with the objectives set out in the national policy, using clearly defined indicators’ (p. 11)^(2)^.

## Methods

The CMAT-R and CMAT-PCT were developed based on a literature review and expert and government consultations. Briefly, the CMAT-R is an app-based tool that collects M2K data from restaurants (including photos), and the CMAT-PCT was developed to assess the content of photos of exterior and interior restaurant advertisements. Methods for the literature review, expert consultations, tool development and tool testing are described below, and results for each component are described in the following section.

### Literature review

A scoping review was undertaken in 2020 to summarise existing evidence on monitoring food and beverage marketing to children and youth in restaurants. Relevant methods and findings from the literature review are described below, and the full scoping review report is available in the supplementary material. The synthesis was written to describe practical measures that could be adopted to monitor food advertising in restaurants that could be seen by children (<13 years old) or teenagers (13–17 years old). In particular, the review sought to examine techniques used to market foods and beverages to children and teens in restaurant settings and key features of published tools that assess food and beverage advertising in restaurant settings.

#### Data sources and search strategy

Searches were completed in August 2020 by a librarian from Health Canada’s Health Library. Concepts related to the research questions were searched in four databases, including Ovid MEDLINE (coverage from 1946 to 2020), EMBASE (coverage from 1974 to 2020), Food Science and Technology Abstracts (coverage from 1969 to 2020) and Global Health (coverage from 1973 to 2020). Databases were selected to be comprehensive and cover a broad range of disciplines related to health, food and nutrition. Concepts that addressed research questions included restaurants, food and beverages, and marketing.

In addition to the academic literature, the National Collaborative on Childhood Obesity Research (NCCOR) food environment measures registry was searched for relevant assessment tools. The NCCOR measures registry is a regularly updated, searchable database of diet and physical activity measures related to childhood obesity research (https://www.nccor.org/nccor-tools/measures/). It contained 367 measures (as of December 2020) related to the food environment. All measures were searched for potentially relevant assessment tools for restaurant environments.

#### Eligibility criteria

Studies were included if they described any tool that assessed some aspect of marketing or advertising within restaurant, were published in English and were published after 2005.

#### Screening

Titles and abstracts of citations were reviewed. Where abstracts were not available, articles were included in the full article review process in the second phase. All citations deemed relevant after the title and abstract screening phase were obtained for a full-text review.

#### Data synthesis

The following data were extracted from each article into separate columns in MS Excel (2016) for coding purposes: study objective, study design, city/region in which the study was conducted, country of the study, population studied, children (<13 years old) addressed specifically, teens (13–17 years old) addressed specifically, type of restaurant (e.g. sit-down and fast food), number of retailers assessed, type of assessment approach, marketing technique assessed (product, price, promotion and placement), validity test of assessment measure, reliability test of assessment measure, tool name, type of outcome assessed, key findings and other relevant information.

### Expert consultations

To assess face validity and improve content validity, four academic experts on food and beverage M2K in Canada were invited to participate in expert consultations, and three participated. Recommendations for expert consultations typically suggest between five and twenty experts should participate to optimise content validity^(36,37)^. In this study, fewer than the recommended number of experts were invited, which was a limitation of the study and was a function of time and resource constraints common to applied research undertaken with external organisation (e.g. governmental agencies). Experts were identified by Health Canada based on M2K expertise (e.g. publishing peer-reviewed academic articles on M2K in Canada) and familiarity with the current state of M2K policy in Canada. Prior to each consultation, experts were provided a summary of findings from the literature review as well as a preliminary draft of the assessment tool. Consultations were facilitated by a semi-structured question guide developed by the research team in collaboration with Health Canada and involved 1–1·5 h video conferences with each of the three experts. The goals of the question guide were to elicit specific feedback on the draft assessment tool and on the findings of the literature review. Detailed notes were recorded during each meeting.

### Tool and user guide development

**Fig. 1:**
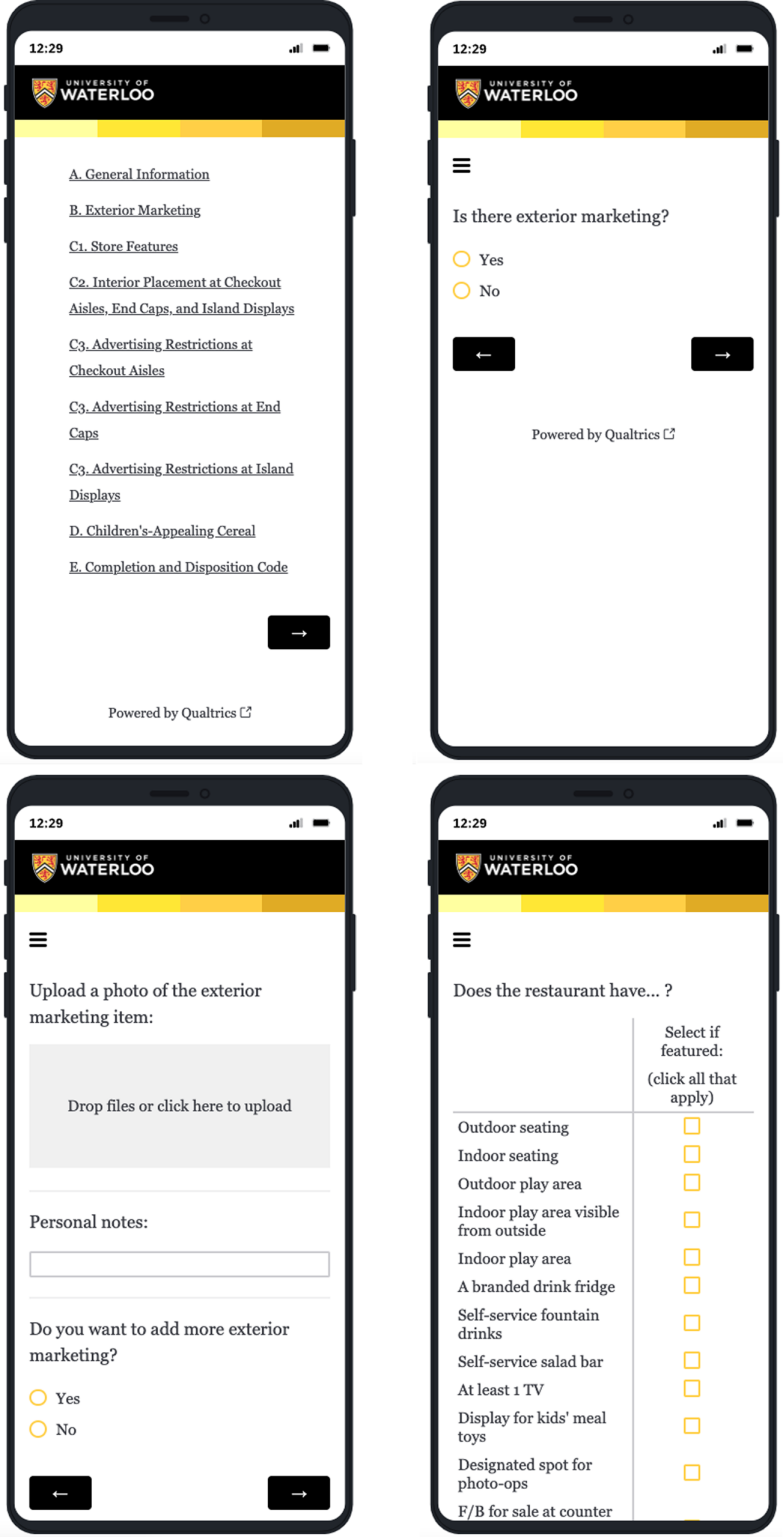
Screenshots of CMAT-R online survey

The CMAT-R was programmed into Qualtrics XM Online Surveys (Qualtrics, Provo, UT) after revising the draft tool according to literature review findings and expert consultation findings (see Fig. 1 for screenshots of the online survey). The first and second authors who collected data for this study downloaded the Qualtrics XM Offline App onto their mobile phones, which allows offline data collection.

A user guide for the CMAT-R was developed to provide clear and specific guidance on completing all measures. Logbooks were created to identify the date of assessment, the restaurant name, location, the restaurant ID (an ID used to identify the restaurant in the Qualtrics survey database) and any notes (e.g. explanatory notes if data collectors could not complete data collection).

The CMAT-PCT was developed as a direct result of the experts’ recommendation to capture photos in the CMAT-R and was created in consultation with the experts and Health Canada. The CMAT-PCT incorporated features of Health Canada’s table of indicators of marketing techniques to be tracked across marketing media and settings as well as the child-appealing packaging coding tool, which measures the presence, type and power of child-appealing marketing on food packaging based on the marketing techniques displayed^(38)^.

Two university-educated coders were trained in the use of the CMAT-PCT by the first and second authors.

### Tool testing

To assess inter-rater reliability of the CMAT-R, a purposefully selected sample of fifty-seven restaurants (fast food, sit-down and buffet-style) were assessed separately by the first and second authors on the same days in the city of Waterloo, Ontario, in August 2021. Restaurants were selected to represent a range of neighbourhoods, including by neighbourhood income and by urban core *v*. suburban developments. To assess inter-rater reliability of the CMAT-PCT, thirty randomly selected photos of interior and exterior restaurant marketing from the fifty-seven assessed restaurants were assessed separately by two trained coders.

### Statistical analysis

To assess inter-rater reliability, Cohen’s Kappa and per cent agreement were calculated for dichotomous variables, and intra-class correlation coefficients (ICCs) were calculated for continuous or rank-order variables. ICCs were calculated using two-way mixed effect model where rater effects were random and measure effects were fixed^(39)^.

Inter-rater reliability data are presented separately by CMAT-R section (general information, drive-thru items, children’s menu items, dollar/value menu items and by CMAT-PCT photo coding results (interior and exterior marketing)). Kappa values >0·80 were considered strong^(40)^. All statistical analyses were conducted in 2022 in SPSS version 28.0.0.0.

## Results

### Scoping review results

**Fig. 2:**
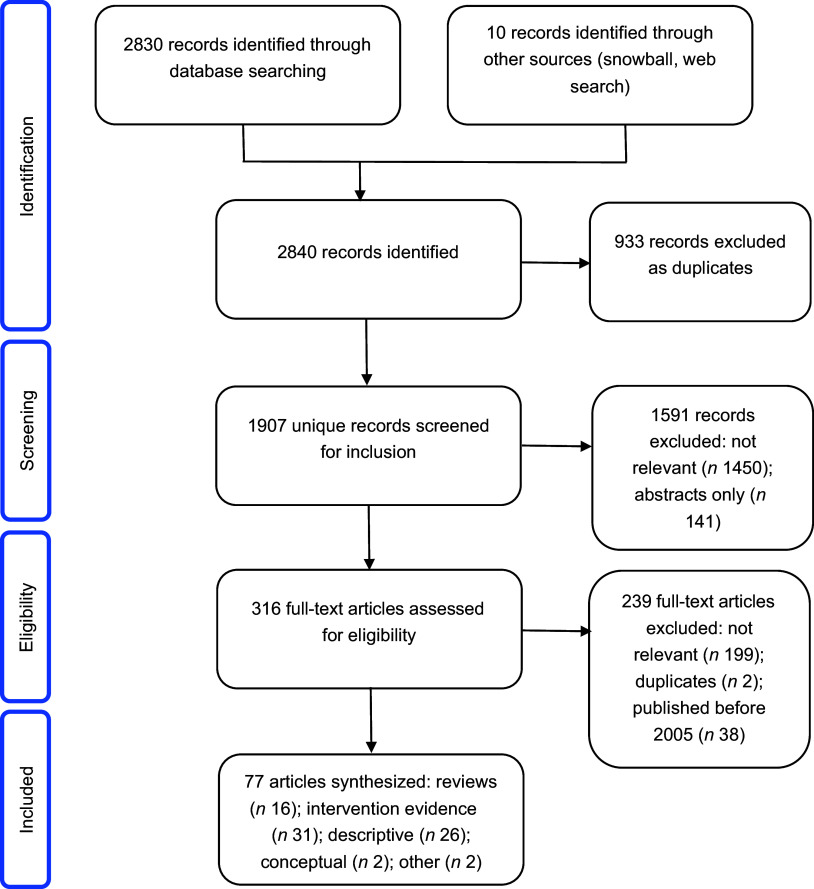
PRISMA flowchart of study selection process

A total of 2840 records were identified (2830 from the academic literature search and ten from other sources), of which 933 were excluded as duplicates in the identification phase. Next, titles and abstracts of 1907 articles were screened for inclusion, of which 1591 were excluded. A total of 316 articles were included in the full-text eligibility screening stage. At this stage, 239 articles were excluded, leaving a total of seventy-seven articles included in the review. Figure 2 shows the Preferred Reporting Items for Systematic reviews and Meta-Analyses (PRISMA) flowchart of the study selection process.

All restaurant assessment tools were published since 2007: fifteen articles described tools assessing indoor restaurant marketing (eight of which also described reliability/validity testing), and eleven articles described tools assessing outdoor restaurant marketing (five of which also described reliability/validity testing).

All indoor and outdoor marketing assessment tools assessed aspects of *product* marketing techniques (e.g. types and/or level of healthiness assigned to food or beverages being advertised). *Promotion* marketing strategies frequently included the type of advertisement (e.g. billboard *v*. bus stop *v*. exterior advertisement on a restaurant), availability of nutrition information, toys given with children’s meal purchases and indoor/outdoor play structures at restaurants. Marketing techniques related to *price* typically included assessing special price promotions, the availability of a ‘dollar’ or ‘value’ menu, the relative cost of ‘healthier’ *v*. ‘less healthy’ options, and the actual price of indicator foods or drinks. Finally, *placement* marketing strategies assessed by reviewed included proximity to schools or low-income areas (for outdoor marketing assessment tools) and placement of unhealthy options at the restaurant counter (for indoor marketing assessment tools).

Four tools stood out as particularly relevant, none of which fully captured policy-relevant data for the current Canadian context in multiple types of restaurants. The NEMS-R^(41)^ assesses the quality and availability of healthy options on children’s menus. The CX3 Tier 2 Fast Food Marketing Environment Survey^(42)^ assesses child-oriented marketing practices such as characters, signs, play areas and toys. The Children’s Menu Assessment^(43)^ examines the nutritional quality of children’s menus in addition to indoor advertising and availability of toys (and was adapted from the NEMS-R). Finally, the child-focused EAT for quick-service restaurants (also adapted from the NEMS-R) assesses advertising in interior locations (e.g. counters and other indoor areas), on the menu board, pricing, exterior signage and promotions. However, the EAT does not assess drive-thru menus or promotions, and it was developed specifically for quick-service restaurants instead of multiple types of restaurants.

Based on the results of the scoping review, a number of recommendations emerged for a restaurant M2K assessment tool for Canada. These recommendations included that the tool be developed to assess: children’s menus, availability of nutrition information, menu indications of ‘healthier options’, dollar/value menus, combo meals, drive-thru menu boards, caloric beverage refill options (e.g. free refills on caloric beverages), availability of toys/gifts with purchase of children’s meals, ‘kids eat free’ or ‘parents eat free’ options, interior displays and signage, and exterior displays and signage.

Based on recommendations emerging from the literature review, and in consultation with Health Canada to ensure that the tool would meet policy-relevant goals, a draft assessment tool was adapted from previously validated tools found in the literature review.

### Expert consultation results

Experts were unsurprised by the literature review findings and noted that restaurant marketing evolves rapidly. Therefore, all three experts suggested capturing additional marketing strategies that had not been captured in prior assessment tools based on their own ongoing research in this area. For example, one expert suggested assessing children’s placemats and colouring activities, discounts for children’s sports teams and restaurant placement (in terms of proximity to schools). Another expert suggested that play structures within restaurants or on restaurant property should be noted. One expert recommended monitoring the extent to which restaurants have mobile phone applications, as these may be particularly appealing to youth (middle-high and high school students). A final recommendation shared by two experts was that the tool be modified from its original version to capture photos, which, in their opinion, could be re-analysed in the future to better capture food industry marketing innovations compared to the series of closed-ended questions proposed in the original tool. The draft tool was revised to reflect the expert recommendations. Specifically, all recommended changes were made with the exception of the recommendation about monitoring mobile phone applications. The reason for this is that Health Canada was aware of other M2K projects aimed at monitoring app-based, online and social media M2K, and monitoring these types of applications was thought to be outside the scope of the current project (which focused on point-of-sale M2K).

### Tool development

The CMAT-R assesses the availability of outdoor seating, play areas (exterior and interior), drive-thrus, indoor seating, items for sale at the counter, displays for kids’ meal toys/giveaways, self-service salad bar and self-service fountain drinks. The availability of nutrition information on both the regular and children’s menu (including a contextual statement), ‘super-size’ options, whether the restaurant accepted loyalty cards or student cards and whether a ‘happy hour’ type of price promotion (where reduced food costs with no required alcohol purchase were offered) were also captured.

Features of both the children’s menu and the dollar or value menu were assessed, given that adolescents tend to be price sensitive and may thus be drawn to value menus^(44)^.

The CMAT-PCT was developed by the authors in consultation with the experts and Health Canada and assessed child-appealing features of exterior and interior advertising using Health Canada’s monitoring food and beverage marketing to children report^(45)^ to align with all current marketing to children monitoring efforts ongoing in Canada. To assess ‘healthfulness’ of food, the tool used Health Canada’s protocol for classifying foods^(46)^ to define products ‘of concern’ from a nutritional standpoint *v*. products ‘not of concern’ from a nutritional standpoint. Briefly, foods and beverages categorised as products ‘not of concern from an advertising perspective’ include products that do not contain free sugars, added sodium or added fat. Examples include plain water, grain flour and unsweetened yogurt. Products ‘of concern from an advertising perspective’ are those that are more likely than not to exceed relevant nutrition thresholds (e.g. total of 2 g saturated fat, 5 g sugar per serving and 140 mg of sodium per serving). Examples include regular soft drinks, sweetened breakfast cereals and flavoured or sweetened yogurts.

### Tool testing results

The first and second authors pre-tested the tool for flow, timing and identifying bugs in the Qualtrics programming in ten restaurants purposefully selected to represent a variety of types of restaurants (e.g. café, fast food outlet, sit-down restaurant, pizza place, ice cream shop and smoothie bar). Modifications were made to the tool based on the flow of the ease of use for data collection. The user guide was updated to reflect all modifications. The CMAT-R took an average of 13 min per restaurant for data collection, and the CMAT-PCT took an average of 5 min per photo to code.

The inter-rater reliability of the CMAT-R tool was strong (mean per cent agreement was 92·4 %, mean Cohen’s κ = 0·82 for all dichotomous variables and mean ICC = 0·961 for continuous/count variables). The per cent agreement, Cohen’s κ and ICC statistics for each item are presented in Table 1. The far left column of Table 1 describes the general characteristics of the evaluated restaurants, which may be used to derive adaptations of our tool for other contexts.

**Table 1:** Inter-rater reliability of the CMAT-R in restaurants (fifty-seven restaurants, Waterloo, Ontario, 2021)

	Item	% agreement	Cohen’s κ
General	Does the restaurant have…		
	a) Outdoor seating?	93	0·835
	b) An outdoor play area?	100	1·000
	c) Indoor play area visible from outside?	100	1·000
	d) A drive-thru?	100	1·000
	e) Third-party delivery service?	90	0·729
	f) Indoor seating?	94	0·788
	g) An interior play area?	100	1·000
	h) A display for kids’ meal toys	100	1·000
	i) Products of concern for sale at the counter?	85	0·664
	j) Self-service salad bar	100	1·000
	k) Self-service fountain drinks	100	1·000
	Is there a regular menu?	100	1·000
	Does the menu contain calorie information?	93	0·844
	Does the menu contain contextual information?	89	0·757
	Is there a super-size option available?	100	1·000
	Are there any promotions for ‘kids eat free’ or ‘parents eat free’?	100	1·000
	Are there any promotions for sports team discounts?	100	1·000
	Is there a ‘happy hour’ with reduced food costs (no alcohol purchase necessary)?	78	0·319
	Is there a student card/loyalty programme accepted?	82	0·599
Drive-thru	Does the drive-thru menu board provide nutritional information?	100	1·000
	If nutritional information is provided on the drive-thru menu for all or some items, what kind of nutrition info?	83	0·714
	Does the drive-thru menu board…?		
	a) Promote at least one food or beverage not of concern?	83	0·714
	b) Promote at least one food or beverage of concern?	100	1·000
	c) State ‘nutrition info available upon request’	100	1·000
	d) Have ads for kids (e.g. toys, camp and sports team promotions)	83	0·714
Children’s Menu	Does the kids’ menu contain…?		
	a) Calorie information	92	0·841
	b) Contextual information	88	0·762
	Does the kids’ menu offer…?		
	a) Combo meals	75	0·400
	b) Activities (colouring, puzzles, games, jokes, etc.)	83	0·667
	c) Free refills on caloric beverages	79	0·571
	d) Links to online games/resources	100	1·000
	e) Free toys/child-oriented giveaways	96	0·864
	f) Included beverages that would be subject to ad restrictions?	91	0·617
	g) Included sides of concern?	83	0·667
	h) Included desserts of concern?	88	0·75
	Count on the kids’ menu…		ICC
	a) Number of entrees of concern	–	0·938
	b) Number of entrees not of concern	–	1·000
	c) Number of sides of concern	–	0·927
	d) Number of sides not of concern	–	0·960
	e) Number of desserts of concern	–	0·914
	f) Number of desserts not of concern	–	0·924
Dollar/value menu	Is there a dollar or value menu?	100	1·000
	Count on the dollar/value menu…		ICCs
	a) Number of entrees of concern	–	0·83
	b) Number of entrees not of concern	–	1·000
	c) Number of sides of concern	–	1·000
	d) Number of sides not of concern	–	1·000
	e) Number of desserts of concern	–	1·000
	f) Number of desserts not of concern	–	1·000

Table 2 shows the inter-rater reliability of CMAT-PCT for restaurant interior and exterior advertisements. The mean per cent agreement for photo coding across items was 97·3 %, and mean Cohen’s κ across items was 0·91, indicating very strong inter-rater reliability.

**Table 2:** Inter-rater reliability of the CMAT-PCT for restaurant interior and exterior advertisements (thirty randomly selected photos from restaurants in Waterloo, Ontario, 2021)

	Item	% agreement	Cohen’s κ
Picture features	Is this an exterior ad?	96·7	0·930
	Is this an interior ad?	96·7	0·930
	Is there a food/beverage featured?	100	1·000
	Number of products featured (0, 1–10, 11+)	100	1·000
	Is a child product featured?	86·7	0·520
Advertisement features	Is a food or beverage brand featured?	96·7	0·902
	Is the price displayed on the ad?	100	1·000
	Is there a price promotion displayed?	100	1·000
	Is a logo or company name present?	96·7	0·902
	Is a slogan present in the ad?	86·7	0·730
	Is there a taste description in the ad?	80·0	0·536
	Is an incentive described (e.g. contest and giveaway)?	96·7	0·930
	Are websites/social media platforms/handles mentioned?	96·7	0·839
	Is a loyalty programme mentioned?	100	1·000
	Is a sense of urgency present?	90·0	0·526
Child/teen appeal	Is a child-directed graphic design present?	100	1·000
	Is a child-directed theme present?	100	1·000
	Is a teen-directed theme present?	100	1·000
	Is a child appeal to ‘fun’ or ‘cool’ present?	100	1·000
	Is a teen appeal to ‘fun’ or ‘cool’ present?	100	1·000
Characters	Is a cartoon present?	96·7	0·930
	Is a child-appealing brand character present?	100	1·000
	Is a child-appealing licenced character present?	100	1·000
	Is a child-appealing celebrity present?	100	1·000
	Is a child actor featured?	100	1·000
	Is a child-appealing cross-promotion present?	100	1·000
	Is a teen-appealing licenced character present?	100	1·000
	Is a teen-appealing celebrity present?	100	1·000
	Is a teen actor featured?	100	1·000
	Is a teen-appealing cross-promotion present?	100	1·000
Other	Is there an appeal to health/nutrition?	90	0·672
	Is there an appeal to product/food convenience?	100	1·000
	Is there an appeal to other benefits?	96·7	0·651
	Is there a gender-based marketing component?	100	1·000

## Discussion

This paper describes the development and reliability testing of the CMAT-R and CMAT-PCT, two related tools designed to assess policy-relevant point-of-sale marketing in restaurants in Canada with a focus on marketing to children and teens. These are the first restaurant-based M2K monitoring tools that capture both interior and exterior, policy-relevant M2K across restaurant types. A major strength is the inclusion of photo data, which can be analysed and re-analysed in the future as food industry marketing innovations emerge. Both tools had high face validity, and while content validity was not formally assessed, the inclusion of items based on a literature review and expert reviewers’ recommendations suggest that content validity was also high. Inter-rater reliability of the CMAT-R was high, with an average per cent agreement of 92·4 % and Cohen’s κ of 0·82 for all dichotomous variables and mean ICC = 0·961 for continuous/count variables. The CMAT-PCT similarly showed excellent inter-rater reliability (mean per cent agreement across items of 97·3 % and mean Cohen’s κ across items of 0·91).

The lack of knowledge around point-of-sale marketing of foods and beverages to children in restaurants in Canada is a critical gap for three reasons. First, purchasing decisions are influenced at the point of sale in restaurants. A recent study from the USA found that 80 % of children 4–16 years old dining at quick-service restaurants selected their meal without parental involvement, and almost 40 % of them decided *in the restaurant* what to eat^(47)^. Moreover, interventions to improve healthy eating in restaurants have been found to improve dietary intake of nutritious foods and reduce fat intake^(48)^, indicating that restaurant cues can in fact influence diet quality. Second, forthcoming national M2K regulations make this knowledge gap especially urgent. Third, as noted above, marketing evolves in response to policies aiming to curtail it. As noted, relevant tobacco industry history demonstrates that as different promotional activities were banned, tobacco companies shifted to different marketing strategies to continue communicating brand imagery^(12–14)^. Indeed, monitoring M2K is critical for both policy development and implementation across settings^(10)^.

There are strengths and limitations associated with the tools and with the current study. With respect to strengths of the study, the process by which the tool was developed included a scoping review, collaboration with Health Canada (the federal department tasked with monitoring food and beverage marketing over time), expert interviews, and pilot testing and modification in a number of different restaurant types. Other tools that assess marketing to children and youth in restaurants (e.g. the EAT^(29)^ and the Bridging the Gap Fast Food Observation Form^(31,32)^) were adapted from previously developed tools, most notably the NEMS-R^(30)^, which was first published in 2007. While both the EAT and the Bridging the Gap tools also used experts to inform their adaptation, neither tool relied on an updated literature synthesis, which was a major strength of the current study. The process undertaken in the current study ensured that the tool would address existing gaps in the literature as well as policy-relevant and cutting-edge research gaps. In terms of strengths of the tools themselves, input from both marketing experts and from Health Canada ensured high face validity and policy relevance, as noted. Both the CMAT-R and the CMAT-PCT showed high inter-rater reliability, which was a strength, as was the fact that the tool has been designed for implementation in multiple types of restaurants. In addition, the tool being available on an app that could be accessed through data collectors’ mobile phones made it less intrusive in terms of data collection than using a clip-board and paper-based copies of the assessment tool. Finally, the CMAT-R’s ability to capture photos was a strength given that the photos can be saved and used for future additional data coding and analysis. This may be especially relevant as food industry develops new marketing tactics in response to evolving restrictions.

Limitations of the study included that the scope was limited to a single municipality in Ontario, Canada. The city of Waterloo is a rapidly growing, mid-sized municipality about 120 km away from Canada’s most populous and diverse city, Toronto. In 2021, 27·5 % of Waterloo’s residents were born outside of Canada^(49)^, which is roughly comparable to the overall Canadian proportion of immigrants. The city’s rapid growth and increasing diversity have resulted in a large variety of diverse restaurant types, including fast food, sit-down and multiple types of ethnic cuisines available. Therefore, despite the study being limited to one municipality, the tools were tested in a variety of different restaurant types. Another potential limitation is that the timing of data collection excluded major holidays, so seasonal or holiday-related promotions may have been missed. Limitations of the tools themselves include the fact that they were not designed to capture other retail sources of foods (e.g. mobile food carts), which may be more relevant for dietary intake in other countries. That said, the CMAT-R and CMAT-PCT are both freely available for adaptation by other researchers, including training manuals and slides, user guides and access to the online app (for researchers whose institutions have a Qualtrics licence) by contacting the first author. A final limitation as described above is that only three experts participated in the expert interviews; recommended practice is between five and twenty experts^(36,37)^. Time and resource constraints precluded more experts from being invited to participate. Future adaptations of these tools would benefit from more fulsome consideration of expert perspectives.

Regarding practical application of the tools, the CMAT-R took approximately 13 min per restaurant to complete, and the CMAT-PCT took approximately 5 min per photo to code. Even after fixing bugs in the Qualtrics program, uploading data to the Qualtrics server was sluggish for data collectors who used the Qualtrics offline version. The offline version of Qualtrics may be preferable for data collectors because of limitations in their data plans (data collectors used their own cell phones). Therefore, to optimise resources needed to implement these tools, future iterations of the CMAT-R may be further modified to focus on the most crucial data to collect and/or try another online platform by which to collect data. Some items captured by the CMAT-R (e.g. a ‘happy hour’ promotion or the ‘dollar’ or ‘value’ menu assessment) may be considered general marketing, rather than child- or teen-specific marketing. These data were captured in the current study because teens tend to be price sensitive and may thus be drawn to value menus^(44)^. However, the tool could be further adapted to only focus on clear instances of marketing to children, rather than items that may also be considered general marketing related to price. In terms of using the CMAT-PCT, future research could explore generative artificial intelligence platforms to code marketing data from photos.

The CMAT-R and CMAT-PCT were developed to monitor restaurant food and beverage marketing in consultation with marketing experts and Health Canada. Both showed high inter-rater reliability and can be adapted to better suit other national contexts. Ongoing M2K monitoring is necessary during policy implementation to ensure compliance and policy enforcement^(10)^. Given the current policy context in Canada, monitoring marketing in restaurants will be an especially important undertaking given the likelihood that point-of-sale restaurant M2K will likely evolve, which has implications for youths’ dietary intake.
